# Supplementary material for: The DNA adenine methylase of Salmonella Enteritidis promotes their intracellular replication by inhibiting arachidonic acid metabolism pathway in macrophages
Source: Front Microbiol. 2023 Mar 2;14:1080851. doi: 10.3389/fmicb.2023.1080851 (PMC10018194; doi:10.3389/fmicb.2023.1080851)
Supplement: Supplementary file 2 [file Data_Sheet_2.zip › S1 Appendix. Non-targeted metabolomics raw data/4.MetDiffAnalysis/C50336_WT.vs.control/C50336_WT.vs.control_neg_heatmap.pdf]

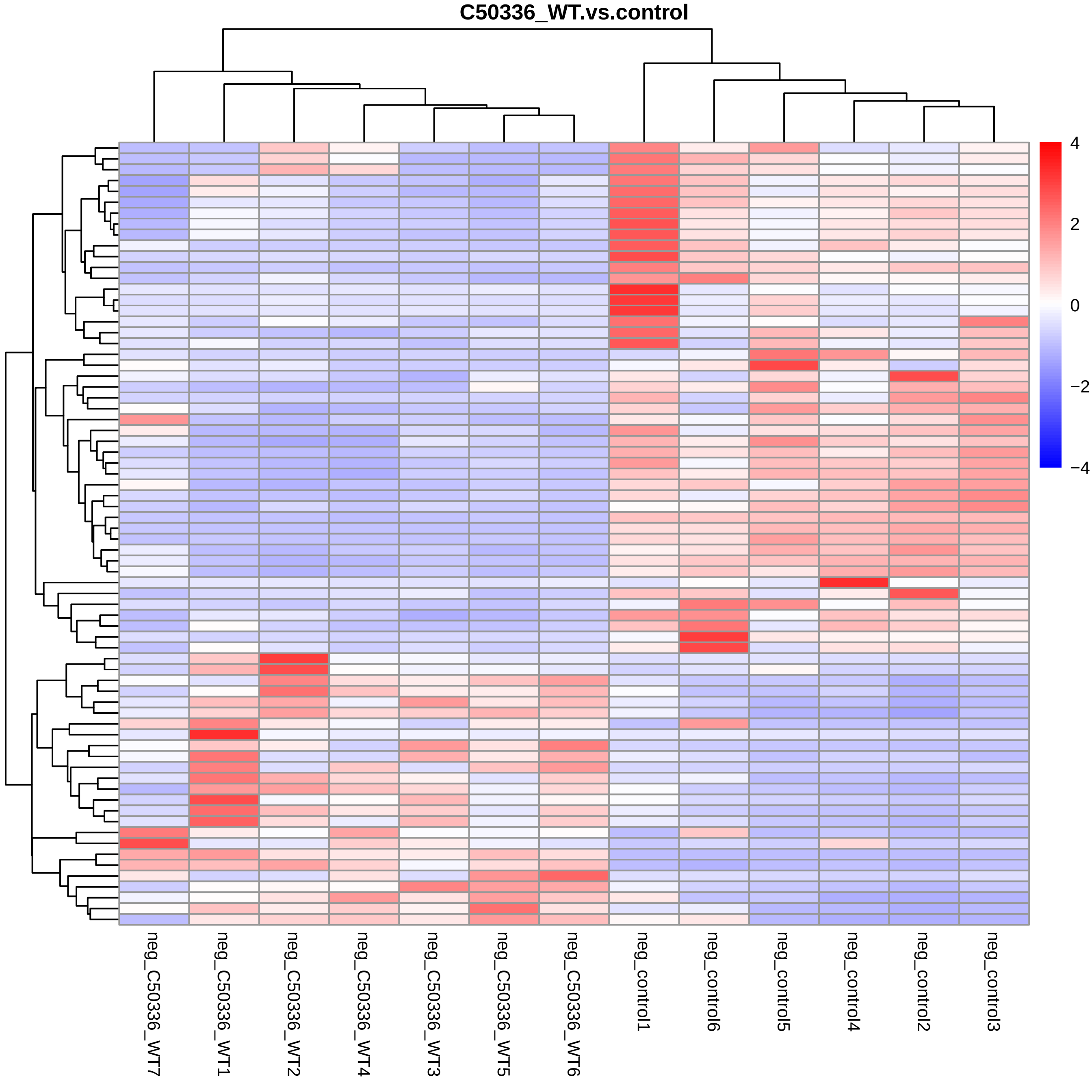
C50336_WT.vs.control
4
2
0
−2
−4
neg_C50336_WT7
neg_C50336_WT1
neg_C50336_WT2
neg_C50336_WT4
neg_C50336_WT3
neg_C50336_WT5
neg_C50336_WT6
neg_control1
neg_control6
neg_control5
neg_control4
neg_control2
neg_control3
